# Effects of triheptanoin (UX007) in patients with long‐chain fatty acid oxidation disorders: Results from an open‐label, long‐term extension study

**DOI:** 10.1002/jimd.12313

**Published:** 2020-09-14

**Authors:** Jerry Vockley, Barbara Burton, Gerard Berry, Nicola Longo, John Phillips, Amarilis Sanchez‐Valle, Kimberly Chapman, Pranoot Tanpaiboon, Stephanie Grunewald, Elaine Murphy, Xiaoxiao Lu, Jason Cataldo

**Affiliations:** ^1^ University of Pittsburgh Pittsburgh Pennsylvania USA; ^2^ Ann & Robert H. Lurie Children's Hospital Chicago Illinois USA; ^3^ Boston Children's Hospital Boston Massachusetts USA; ^4^ University of Utah Salt Lake City Utah USA; ^5^ Vanderbilt University Medical Center Nashville Tennessee USA; ^6^ USF Health Morsani College of Medicine Tampa Florida USA; ^7^ Children's National Health System Washington District of Columbia USA; ^8^ Great Ormond Street Hospital and Institute of Child Health NIHR Biomedical Research Center (BRC), UCL London UK; ^9^ National Hospital for Neurology and Neurosurgery London UK; ^10^ Ultragenyx Pharmaceutical Inc. Novato California USA

**Keywords:** cardiomyopathy, long‐chain fatty acid oxidation disorders (LC‐FAOD), rhabdomyolysis, triheptanoin (UX007)

## Abstract

Long‐chain fatty acid oxidation disorders (LC‐FAOD) are autosomal recessive conditions that impair conversion of long‐chain fatty acids into energy, leading to significant clinical symptoms. Triheptanoin is a highly purified, 7‐carbon chain triglyceride approved in the United States as a source of calories and fatty acids for treatment of pediatric and adult patients with molecularly confirmed LC‐FAOD. CL202 is an open‐label, long‐term extension study evaluating triheptanoin (Dojolvi) safety and efficacy in patients with LC‐FAOD. Patients rolled over from the CL201 triheptanoin clinical trial (rollover); were triheptanoin‐naïve (naïve); or had participated in investigator‐sponsored trials/expanded access programs (IST/other). Results focus on rollover and naïve groups, as pretreatment data allow comparison. Primary outcomes were annual rate and duration of major clinical events (MCEs; rhabdomyolysis, hypoglycemia, and cardiomyopathy events). Seventy‐five patients were enrolled (24 rollover, 20 naïve, 31 IST/other). Mean study duration was 23.0 months for rollover, 15.7 months for naïve, and 34.7 months for IST/other. In the rollover group, mean annualized MCE rate decreased from 1.76 events/year pre‐triheptanoin to 0.96 events/year with triheptanoin (*P* = .0319). Median MCE duration was reduced by 66%. In the naïve group, median annualized MCE rate decreased from 2.33 events/year pre‐triheptanoin to 0.71 events/year with triheptanoin (*P* = .1072). Median MCE duration was reduced by 80%. The most common related adverse events (AEs) were diarrhea, abdominal pain/discomfort, and vomiting, most mild to moderate. Three patients had serious AEs (diverticulitis, ileus, rhabdomyolysis) possibly related to drug; all resolved. Two patients had AEs leading to death; neither drug related. Triheptanoin reduced rate and duration of MCEs. Safety was consistent with previous observations.

## Graphical abstract

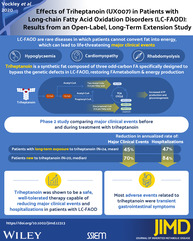

**Synopsis**

Triheptanoin was associated with a reduction in rate and duration of major clinical events in patients with LC‐FAOD.

## 1 INTRODUCTION

Long‐chain fatty acid oxidation disorders (LC‐FAOD) are a group of rare, life‐threatening, autosomal recessive disorders caused by defects in the mitochondrial carnitine shuttle or β‐oxidation enzymes involved in the conversion of dietary long‐chain fatty acids into energy.^1^, ^2^ There are six LC‐FAOD types; three include inherited deficiencies of enzymes required for β‐oxidation: very‐long‐chain acyl‐coenzyme A (CoA) dehydrogenase, long‐chain 3‐hydroxyacyl‐CoA dehydrogenase (LCHAD), and trifunctional protein (TFP) deficiency.^3^ The remaining three include proteins required for long‐chain fatty acid transport: carnitine palmitoyltransferase I and II and carnitine‐acyl‐carnitine translocase.

Symptoms of LC‐FAOD are heterogeneous, especially impacting organs that require high energy production from fatty acid oxidation (FAO), including skeletal muscle, heart, and liver. As a consequence, patients with LC‐FAOD experience rhabdomyolysis, hypotonia, muscle weakness and pain, exercise intolerance, cardiomyopathy, arrhythmia, hypoglycemia, steatohepatitis, and hepatomegaly.^1^, ^2^, ^4^ Complications and symptoms often result in hospitalizations, emergency room visits, or emergency home interventions. Clinical manifestations can be exacerbated during physiological stress due to increased energy needs, and patients with LC‐FAOD and their caregivers show decreased health‐related quality of life.^5^, ^6^, ^7^ Mortality rates have been reported as high as 90% but are reduced in patients identified with newborn screening, suggesting a benefit for early disease management.^8^, ^9^

Dietary management is the current standard of care, with guidelines recommending avoidance of fasting, a low‐fat/high carbohydrate diet, and consumption of even‐carbon medium‐chain triglycerides (MCT) to bypass the defect in long‐chain fatty acid metabolizing enzymes.^10^ Expert dietitians assess each patient's energy and nutrient needs based on age, sex, weight, activity level, and clinical manifestations. Calorie intake provided by protein, carbohydrate, and fat are monitored and adjusted as required to meet changing patient needs. Although not formally studied in a controlled trial, limited improvements can be seen for some patients with dietary management.^7^, ^11^

Triheptanoin (Dojolvi) is a highly purified, GMP grade, synthetic odd‐carbon medium‐chain triglyceride that is a unique energy substrate replacement therapy for patients with LC‐FAOD. Triheptanoin is approved in the United States as a source of calories and fatty acids for the treatment of pediatric and adult patients with molecularly confirmed LC‐FAOD. Upon ingestion, triheptanoin is rapidly broken down into free heptanoate and metabolized by short‐ and medium‐chain FAO enzymes, bypassing the enzymes deficient in LC‐FAOD. Heptanoate is then metabolized into acetyl‐CoA and propionyl‐CoA. Acetyl‐CoA can enter the tricarboxylic acid (TCA) cycle for ATP production or is diverted to the liver for ketogenesis. Propionyl‐CoA is further metabolized to succinyl‐CoA, which resupplies TCA cycle intermediates (anaplerosis) that may be secondarily deficient in patients with LC‐FAOD, ultimately providing energy for gluconeogenesis to increase blood glucose and liver glycogen stores.^12^, ^13^, ^14^, ^15^

In the CL201 phase 2 study (NCT01886378) in patients with LC‐FAOD, triheptanoin reduced the incidence and frequency of hospital days due to major clinical events (MCEs, ie, rhabdomyolysis, hypoglycemia, and cardiomyopathy) and had a positive impact on exercise tolerance, physical health, and vitality.^7^, ^16^ Here, we report interim results from CL202, an open‐label, extension study evaluating triheptanoin in patients with LC‐FAOD to examine long‐term effects of triheptanoin and replicate results in a naïve population.

## 2 METHODS

### 2.1 Design and participants

CL202 (NCT02214160) is an ongoing, open‐label, phase 2 study assessing the efficacy and safety of triheptanoin in patients with LC‐FAOD. This study is being conducted in 10 centers, 8 in the United States and 2 in the United Kingdom. Three cohorts of patients were enrolled: (a) 24 patients rolling over from the phase 2 triheptanoin study CL201 (CL201 rollover group); (b) 20 patients who had failed to improve with current dietary guidelines and had never received triheptanoin (triheptanoin‐naïve group); and (c) 31 patients who participated in an investigator‐sponsored trial or compassionate use program with triheptanoin (IST/other group; NCT01461304, NCT01379625, and NCT01461304) (Figure S1). Eligible patients were at least 6 months of age with a confirmed diagnosis of LC‐FAOD (additional eligibility criteria are available in the supplementary materials).

The 18‐month period prior to the first dose of triheptanoin (or from birth for patients <18 months of age) was used to establish an internal control for each patient in the 201 rollover and triheptanoin‐naïve groups. For these patients, medical records were retrospectively reviewed to document LC‐FAOD history, treatment, and MCEs. Internal controls were not available for the IST/other group.

Patients will receive triheptanoin treatment for up to 5 years, or until commercially available. Triheptanoin was administered orally at a target of 25% to 35% of total daily caloric intake based on tolerability and metabolic energy needs. By design, daily caloric intake with MCT was ~10% lower than daily caloric intake with triheptanoin. Triheptanoin dosing outside this range was permitted if the patient was tolerating a different dose prior to enrollment. The only protocol‐specified dietary recommendation was a change in medium fat chain dose (usually MCT) while maintaining an isocaloric diet; all other macronutrients remained in line with standard guidelines. If a patient was triheptanoin‐naïve or was off triheptanoin for ≥1 month, dosing was titrated to reach the target dose range. Patients switching from MCT to triheptanoin transitioned at the same dose; triheptanoin then was titrated per investigator discretion.

### 2.2 Assessments

Baseline was week 0 for patients in the triheptanoin‐naïve and IST/other groups. For patients in the CL201 rollover group, assessments conducted at the last CL201 visit (week 78) were used for baseline data to avoid duplication. Patients visited the clinic at baseline and every 6 months and received follow‐up telephone calls between clinic visits. Additionally, all patients received an initial safety follow‐up telephone call 2 weeks and 3 months after their first dose of triheptanoin in this study and received additional safety follow up as needed. A safety follow‐up phone call was conducted 30 to 35 days after last dose of study drug. Patients and/or caregivers completed a daily diet diary for 3 days prior to each post‐baseline clinic visit which was reviewed by site staff to calculate daily caloric intake. Adverse events (AEs), MCEs, concomitant medications, and triheptanoin treatment compliance were monitored at all visits. The full schedule of events is available in the supplemental materials.

The primary endpoint was annualized total MCE rate. MCEs were defined as hospitalizations, emergency room visits, or emergency home interventions with a diagnosis of rhabdomyolysis, hypoglycemia, or cardiomyopathy. MCEs were identified through reporting by patients or caregivers at clinic visits and telephone calls. MCEs were confirmed with medical record review and source document verification. Each incidence of rhabdomyolysis, hypoglycemia, or cardiomyopathy was counted as an individual event. Event severity was not captured. Rhabdomyolysis, hypoglycemia, and cardiomyopathy were selected because these are life threatening events that are common across types of LC‐FAOD. The annualized MCE event rate and duration was defined as follows:

$$\mathrm{MCE}\;\text{event rate}=\frac{\text{Total number of MCEs}}{\text{Duration of data collection period}\;\left(\text{in days}\right)/365.25}$$

$$\mathrm{MCE}\;\text{duration}=\frac{\text{Total duration of MCEs}\;\left(\text{in days}\right)}{\text{Duration of data collection period}\;\left(\text{in days}\right)/365.25}$$

Safety was assessed by the frequency and severity of AEs, vital signs, concomitant medications, cardiac function with echocardiograms, and standard safety labs. Rhabdomyolysis was captured as both an MCE and AE. Functional disability and cognitive development were assessed using the physical and mental health component summary scores of the SF‐10 or SF‐12v2, depending on patient age.^17^, ^18^

### 2.3 Analysis

Statistical analyses were mostly descriptive. For the analysis of MCEs, comparisons were performed between pre‐triheptanoin and with‐triheptanoin periods using a paired *t*‐test. When the normality assumption was questionable, median values were presented instead of means, and the Wilcoxon signed‐rank test (WSRT) was performed as a nonparametric alternative to the paired *t*‐test.

Comparisons of MCE frequency and duration between pre‐triheptanoin and with‐triheptanoin periods were also performed using a negative binomial regression model (NBR) to account for different follow‐up times during either pre‐triheptanoin (for infants with <18 months retrospective data collection) or with triheptanoin (for patients who discontinued the study early prior to completing 18 months). The ratio of the event rate with triheptanoin over pre‐triheptanoin was provided, along with the two‐sided 95% CI and *P*‐value. All statistical tests were two‐sided, at a significance level of .05. SAS software version 9.4 was used to perform analyses.

## 3 RESULTS

All six LC‐FAOD were represented among the 75 enrolled patients (Table 1). No differences in LC‐FAOD type were seen by age. Medical history for all patients reflected commonly reported LC‐FAOD manifestations including rhabdomyolysis (84.0%), muscle pain (72.0%), hypoglycemia (61.3%), exercise intolerance (44.0%), cardiomyopathy (41.3%), muscle weakness (36.0%), and failure to thrive (33.3%). Most patients were diagnosed during infancy or early childhood (median age of diagnosis: 0.09 years, range: 0.0‐56.7 years). At the time of this interim analysis (cutoff June 1, 2018), mean duration in CL202 for each group was as follows: CL201 rollover 23.1 months (excludes CL201), triheptanoin‐naïve 15.7 months, and IST/other 34.8 months. Results for the CL201 rollover group compare the 18‐month pre‐triheptanoin period to the 36‐month with‐triheptanoin period (18 months from CL201 and 18 months from CL202). Results for the triheptanoin‐naïve group compare the 18‐month pre‐triheptanoin period to the 18‐month with‐triheptanoin period.

**TABLE 1:** Baseline characteristics

Characteristic	n (%)			
	CL201 rollover n = 24	Triheptanoin‐naïve n = 20	IST/other n = 31	Total n = 75
Age group				
0‐1 y	0 (0.0)	3 (15.0)	0 (0.0)	3 (4.0)
>1‐6 y	9 (37.5)	6 (30.0)	4 (12.9)	19 (25.3)
>6‐18 y	9 (37.5)	7 (35.0)	16 (51.6)	32 (42.7)
>18 y	6 (25.0)	4 (20.0)	11 (35.5)	21 (28.0)
Male	14 (58.3)	12 (60.0)	13 (41.9)	39 (52.0)
Race				
White	21 (87.5)	16 (80.0)	29 (93.5)	66 (88.0)
Asian	1 (4.2)	1 (5.0)	0 (0.0)	2 (2.7)
Black or African American	1 (4.2)	2 (10.0)	1 (3.2)	4 (5.3)
Other	1 (4.2)	1 (5.0)	1 (3.2)	3 (4.0)
LC‐FAOD deficiency type				
VLCAD	9 (37.5)	6 (30.0)	11 (35.5)	26 (34.7)
LCHAD	9 (37.5)	6 (30.0)	9 (29.0)	24 (32.0)
CPT II	3 (12.5)	2 (10.0)	6 (19.4)	11 (14.7)
TFP	3 (12.5)	3 (15.0)	4 (12.9)	10 (13.3)
CACT	0 (0.0)	2 (10.0)	1 (3.2)	3 (4.0)
CPT I	0 (0.0)	1 (5.0)	0 (0.0)	1 (1.3)
Prior treatment with MCT	23 (95.8)^a^	18 (90.0)	N/A	—

Abbreviations: CACT, carnitine‐acyl‐carnitine translocase; CoA, coenzyme A; CPT, carnitine palmitoyltransferase; LC‐FAOD, long‐chain fatty acid oxidation disorder; LCHAD, long‐chain 3‐hydroxyacyl‐CoA dehydrogenase; MCT, medium‐chain triglyceride; TFP, trifunctional protein; VLCAD, very‐long‐chain acyl‐CoA dehydrogenase.

^a^ Prior treatment with MCT before enrollment in CL201.

### 3.1 Annualized MCE rates

**FIGURE 1:**
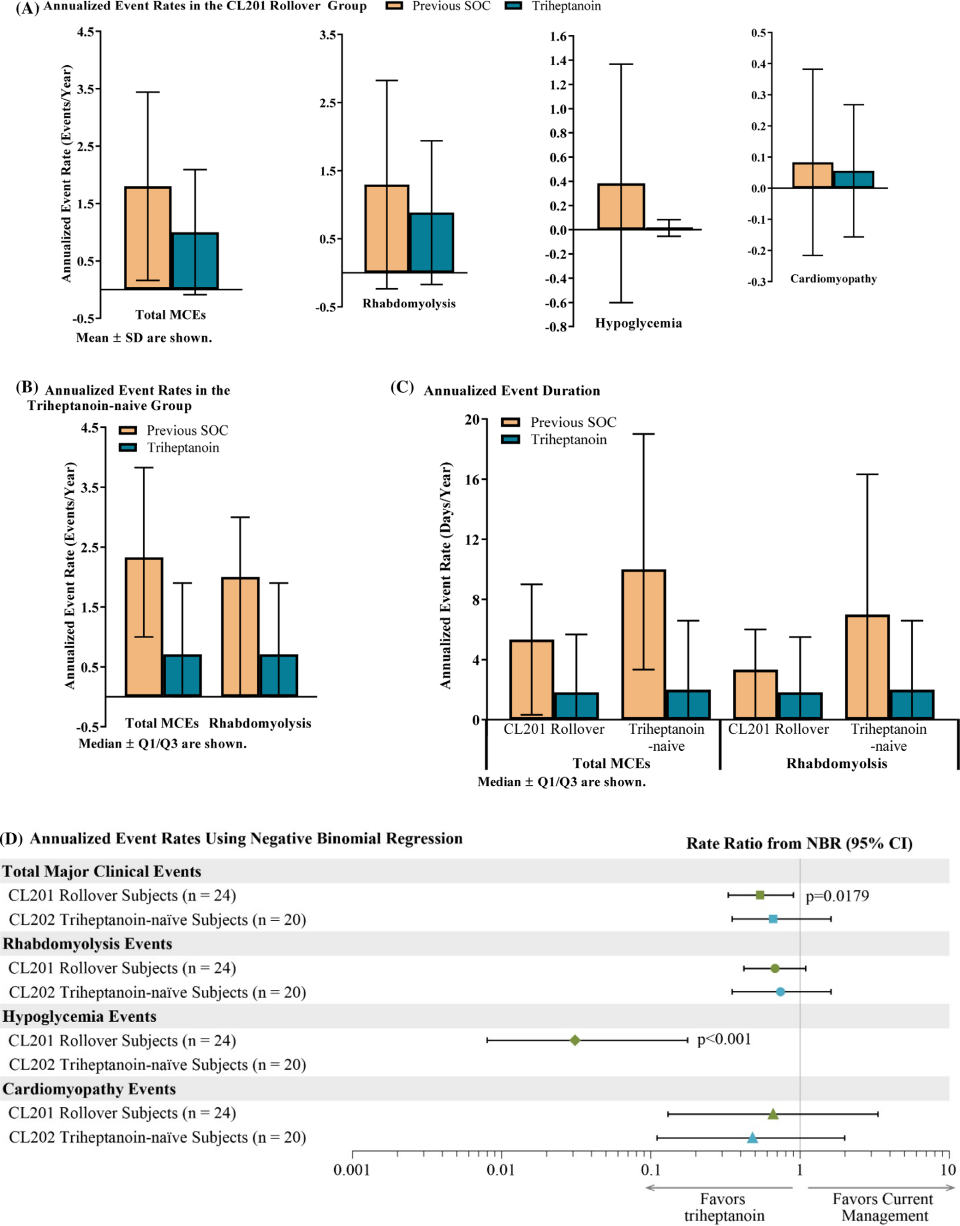
Reduction in major clinical events (MCEs) with triheptanoin. Significant *P*‐values provided on the graph. Event rate (triheptanoin‐naïve) and duration (both groups) for hypoglycemia and cardiomyopathy were <1 events/year and days/year for both the pre‐triheptanoin and with‐triheptanoin periods, and, therefore, were not graphed. In the CL201 rollover group, the with‐triheptanoin period is comprised of the first 36 months following triheptanoin initiation in studies CL201 and CL202, or from triheptanoin initiation to discontinuation for patients who discontinued. In the triheptanoin‐naïve group, the with‐triheptanoin period is the first 18 months following triheptanoin initiation during CL202, or from triheptanoin initiation to discontinuation for patients who discontinued

In the CL201 rollover group, mean (SD) annualized total MCE rate decreased 45%, from 1.76 (1.64) events/year pre‐triheptanoin to 0.96 (10.9) events/year with triheptanoin (Figure 1A; *P* = .0319 paired *t*‐test; median: from 1.53 to 0.50). For MCE event type, mean annualized event rate decreased 32% for rhabdomyolysis (mean [SD] events/year: 1.30 [1.53] pre‐triheptanoin to 0.89 [1.05] with triheptanoin), 96% for hypoglycemia (0.383 [0.98] to 0.014 [0.07]), and 33% for cardiomyopathy (0.083 [0.30] to 0.056 [0.21]). More specifically for hypoglycemia, 12 events occurred in four patients during the pre‐triheptanoin period and only one event occurred during the with‐triheptanoin period on the patient's first day of triheptanoin treatment in CL201.^16^ No hypoglycemia events occurred in the CL201 rollover group during CL202. No marked differences were seen within age groups Supplemental Table S1).

In the triheptanoin‐naïve group, median annualized total MCE rate decreased 70%, from 2.33 events/year pre‐triheptanoin to 0.71 events/year with triheptanoin (Figure 1B; *P* = .1072 WSRT; mean [SD]: from 2.95 [3.010] to 7.72 [27.05]). For MCE event type, median annualized event rate for rhabdomyolysis decreased 65%, from 2.00 events/year pre‐triheptanoin to 0.71 events/year with triheptanoin (*P* = .2734 WSRT). Median annualized event rate for hypoglycemia and cardiomyopathy events were 0 during the pre‐triheptanoin and with‐triheptanoin periods.

Rates for annualized total MCE events and specific event types were similar using NBR (NBR; annualized rate ratio, Figure 1D). In the CL201 rollover group, mean total MCE rate decreased from 1.8 events/year pre‐triheptanoin to 1.0 events/year with triheptanoin (annualized event rate ratio: 0.54, 95% CI: 0.33, 0.90, *P* = .0179). In the triheptanoin‐naïve group, mean MCE rate decreased from 2.3 events/year pre‐triheptanoin period to 1.5 events/year with triheptanoin (NBR; annualized rate ratio: 0.66, 95% CI: 0.32, 1.37, *P* = .2622). Although all specific event types decreased with triheptanoin, only the reduction in hypoglycemia annualized event rate in the CL201 rollover group was significantly decreased (rate ratio 0.04; *P* < .0001).

### 3.2 Annualized MCE duration

In the CL201 rollover group, median annualized total MCE duration was reduced by 66%, from 5.33 days/year pre‐triheptanoin to 1.83 days/year with triheptanoin (*P* = .3002 WSRT; Figure 1C). By NBR, change in mean annualized total MCE event days was not significant (6.4 days/year pre‐triheptanoin and 5.8 days/year with triheptanoin; *P* = .8205). Median annualized duration for rhabdomyolysis was reduced by 45.0%, from 3.33 days/year pre‐triheptanoin to 1.83 days/year with triheptanoin (*P* = .7086 WSRT). By NBR, mean values were 4.0 days/year pre‐triheptanoin and 5.1 days/year with triheptanoin (*P* = .5753). Event duration for cardiomyopathy and hypoglycemia were too small pre‐triheptanoin and with triheptanoin for a sufficient comparison.

In the triheptanoin‐naïve group, median annualized total MCE duration was reduced by 80%, from 10.0 days/year pre‐triheptanoin to 2.0 days/year with triheptanoin (Figure 1C; *P* = .1475; WSRT). By NBR, mean annualized total MCE duration was 17.1 days/year pre‐triheptanoin and 16.2 days/year with triheptanoin (*P* = .9230). For MCE event type, median annualized rhabdomyolysis duration was reduced by 71.4%, from 7.00 days/year pre‐triheptanoin to 2.00 days/year with triheptanoin (*P* = .3335 WSRT). By NBR, mean values were 10.7 days/year pre‐triheptanoin and 15.7 days/year with triheptanoin (*P* = .4206). Event duration for cardiomyopathy and hypoglycemia were too small pre‐triheptanoin and with triheptanoin for a sufficient comparison.

### 3.3 Annualized hospitalization rates

In the CL201 rollover group, annualized hospitalization event rates significantly decreased by 46.9%, from a mean (SD) rate of 1.43 (1.32) events/year pre‐triheptanoin to 0.76 (1.01) events/year with triheptanoin (*P* = .0429 paired *t*‐test). Median annualized hospitalization duration was reduced by 62.1%, from 4.83 days/year pre‐triheptanoin to 1.83 days/year with triheptanoin (*P* = .4390 WSRT).

In the triheptanoin‐naïve group, median annualized hospitalization event rate decreased by 83.5%, from 2.00 events/year pre‐triheptanoin to 0.33 events/year with triheptanoin (*P* = .2764 WSRT). Median annualized hospitalization duration was reduced by 86.2%, from 9.66 days/year pre‐triheptanoin to 1.33 days/year with triheptanoin (*P* = .2918 WSRT).

### 3.4 IST/other

Results for the IST/other group are summarized descriptively, and no comparisons were done. Mean (SD) annualized MCE rate with triheptanoin was 1.92 (3.413) (median [Q1, Q3]: 0.67 [0, 2.14]). Mean (SD) annualized MCE duration with triheptanoin was 9.13 (18.50) (median [Q1, Q3]: 3.18 [0, 7.48]).

### 3.5 Physical functioning and mental health

**FIGURE 2:**
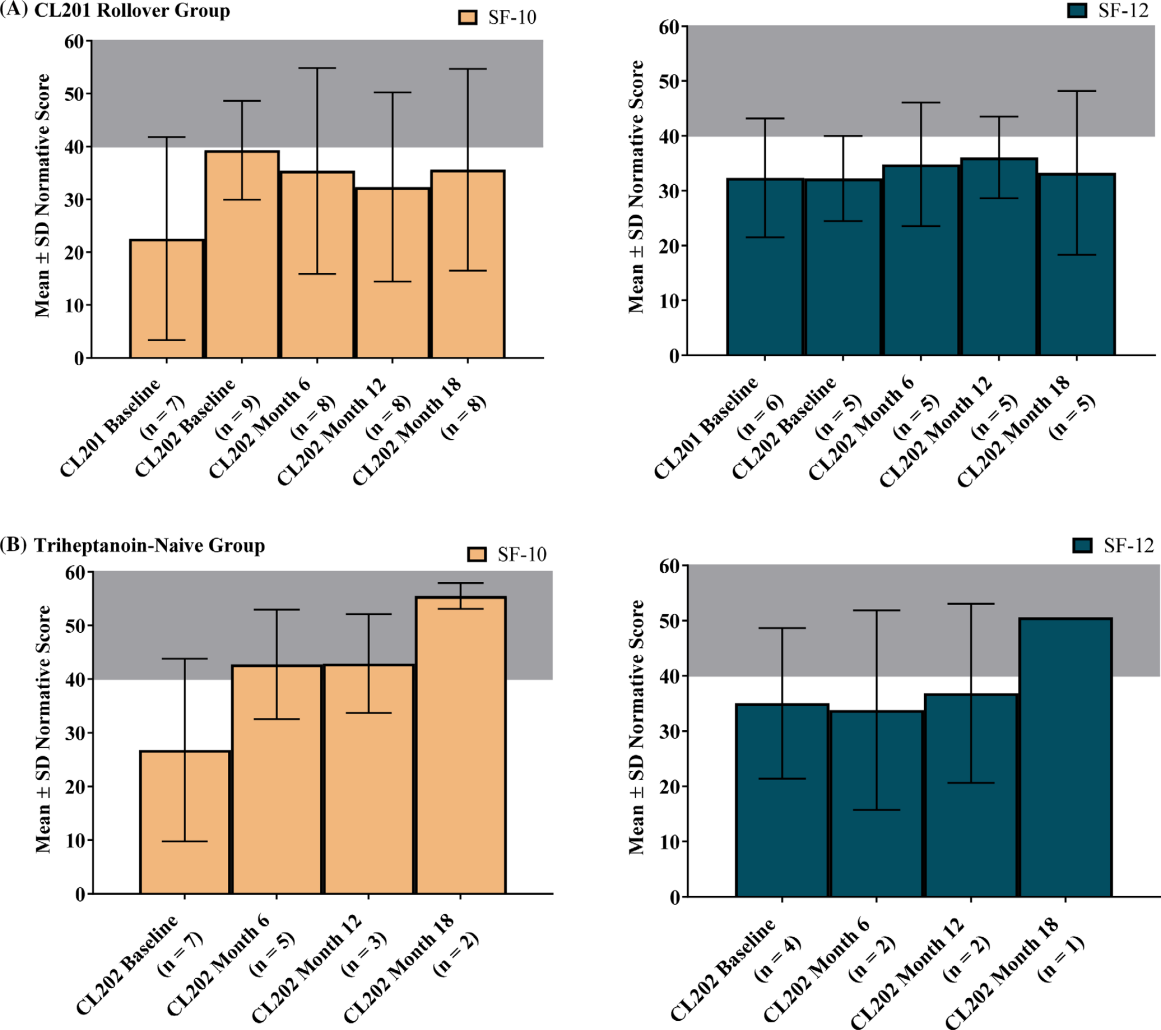
Improvement in physical functioning with triheptanoin. Shaded gray area indicates normal range (40‐60)

Both the CL201 rollover and triheptanoin‐naïve groups showed normative scores below the healthy reference range (40‐60) on the SF‐10 and SF‐12v2 Physical Health Summary (Figure 2). The CL201 rollover group maintained improvements in the Physical Health Summary score observed in the CL201 study, and the triheptanoin‐naïve group replicated improvements observed in the CL201 study. Psychosocial Summary Scores are available in the supplemental appendix.

### 3.6 Safety reporting across all groups

For patients in the CL201 rollover group, safety data are reported for CL202 only. Safety data from CL201 are summarized in Vockley et al.^16^ Across all groups, a majority of patients experienced ≥1 AE, with most AEs grade 1 (mild), 2 (moderate), or 3 (severe) in severity (Table 2). Approximately 49% of patients experienced a severe AE of rhabdomyolysis, all but one of these AEs was considered unrelated to triheptanoin (described below). The most commonly reported AEs were characteristic of the underlying disease: rhabdomyolysis (61% of patients overall), upper respiratory tract infection (URI; 51%), viral URI (37%), vomiting (35%), and diarrhea (31%). Thirty‐four (47%) patients experienced AEs considered at least possibly related to triheptanoin by the investigator; most were gastrointestinal events. Nine patients had a protocol‐specified dose reduction (ranging from a change of 1%‐8% daily caloric intake) to manage gastrointestinal symptoms.

**TABLE 2:** Safety summary for CL202

Category, n (%)	CL201 rollover (N = 24)	Triheptanoin‐naïve (N = 20)	IST/other (N = 31)	Total (N = 75)
AEs	24 (100.0)	19 (95.0)	31 (100.0)	74 (98.7)
Treatment‐related AEs	8 (33.3)	14 (70.0)	13 (41.9)	35 (46.7)
GI‐related AEs	6 (25.0)	12 (60.0)	12 (38.7)	31 (41.3)
Grade 3/4 AE	14 (58.3)	12 (60.0)	23 (74.2)	49 (65.3)
Serious AEs	18 (75.0)	15 (75.0)	24 (77.4)	57 (76.0)
Related serious AEs	1 (4.2)	1 (5.0)	1 (3.2)	3 (4.0)
AEs leading to treatment discontinuation	0 (0.0)	1 (5.0)	0 (0.0)	1 (1.3)
AEs leading to study discontinuation	0 (0.0)	0 (0.0)	0 (0.0)	0 (0.0)
Deaths	0 (0.0)	1 (5.0)	1 (3.2)	2 (2.7)

Abbreviations: AEs, adverse events; GI, gastrointestinal.

There were two deaths, considered unrelated to treatment, in patients with the TFP type of LC‐FAOD, worsening cardiomyopathy in a 4‐year‐old patient in the IST/other group who had been on triheptanoin for 2 years and fatal cardiorespiratory arrest in a 9 month‐old patient in the triheptanoin‐naïve group who had been on triheptanoin for 3 months (additional details in supplemental appendix).

Fifty‐seven (76%) patients experienced a serious AE. Most were hospitalizations for rhabdomyolysis (56.0%, a complication of the underlying disease) or acute infectious disease (46.7%). Three patients had serious AEs considered at least possibly related to triheptanoin by the investigator: diverticulitis (severe), ileus (moderate), and rhabdomyolysis (severe); all resolved. One patient discontinued treatment due to a mild AE of rhabdomyolysis experienced on the first day of treatment with triheptanoin.

There were no noteworthy changes in vital signs, echocardiograms, or routine hematology, chemistry, urinalysis laboratory values throughout the trial.

## 4 DISCUSSION

This study demonstrated that treatment with triheptanoin was associated with reductions in MCE rate and duration and with improvements in physical health. For patients in the CL201 rollover group, continued treatment with triheptanoin resulted in sustained improvements.^7^, ^16^ For patients in the triheptanoin‐naïve group, improvements were consistent with outcomes in CL201. Improvements in physical functioning combined with fewer MCEs and fewer hospitalizations likely contributed to improved quality of life.

The safety profile of triheptanoin in this study was consistent with the safety profile observed in CL201, with similar rates of rhabdomyolysis and infections.^7^, ^16^ No new safety findings were observed. There were two deaths in this study, unrelated to triheptanoin, due to progressive cardiac symptoms in patients with TFP type of LC‐FAOD. Although advances in newborn screening have improved mortality rates, TFP is a more severe type of LC‐FAOD, with patients dying within the first few weeks of life.^19^ Although there was some improvement in cardiac function following the initiation of triheptanoin in a patient who died from cardiac symptoms, the heart relies on long chain fats for 60% to 70% of the required energy supply,^20^ making depletions in energy difficult to overcome.

The results of this clinical trial are consistent with findings from previous triheptanoin studies.^7^, ^16^, ^21^, ^22^ Results from the previous triheptanoin study CL201 showed a 48.1% reduction in mean annualized total MCE event rate and a 50.3% reduction in mean annualized total MCE event duration (days/year) compared to an 18‐month pre‐triheptanoin period. In this extension study, reductions were sustained in the CL201 rollover group (45.5% mean reduction in event rate and 65.7% median reduction in total MCE duration) over an additional 18 months (36 months total) with triheptanoin. The triheptanoin‐naïve group also showed a consistent reduction in annualized total MCE event rate (69.5% median reduction) and duration (80% median reduction). Decreases in hospitalizations and cardiomyopathy events observed in this study were also consistent with previous observational studies in patients with LC‐FAOD receiving triheptanoin through a compassionate use program.^21^, ^22^ Interpretation of changes in cardiomyopathy is limited due to the small number of events experienced in the pre‐triheptanoin period. While this extension study was not specifically designed to detect significant changes in clinical events in triheptanoin‐naïve patients, the trend for both total and specific clinical events mirrored those in other studies, adding to the growing evidence of improvement with triheptanoin for patients with LC‐FAOD. Similarly, the continued trend of improved clinical course in patients transitioning from the previous triheptanoin study suggests that the effect of triheptanoin is long lasting.

Triheptanoin treatment resulted in a near elimination of hypoglycemia events in this trial and previous triheptanoin studies.^16^, ^21^ Reduction in hypoglycemia events likely reflects the unique attribute of triheptanoin as an anaplerotic odd‐carbon medium chain fat energy replacement. With triheptanoin, both acetyl‐CoA and propionyl‐CoA are supplied to the TCA cycle through anaplerosis to increase energy production, and the increase in energy production allows production of glucose and repletion of glycogen stores through gluconeogenesis. Enhanced gluconeogenesis sustains blood glucose levels during periods of metabolic stress such as illness in patients with LC‐FAOD. This mechanism of action also accounts for the reduction in rhabdomyolysis and cardiomyopathy, as heart and muscle rely heavily on long chain fats for energy.^20^

Several limitations may be present. Due to the rare nature of these diseases, a placebo control arm on standard dietary treatment with MCT was not included. Instead, historical pretreatment data served as reference for improvement. The sustained benefit observed in the CL201 rollover group and replication of results in the triheptanoin‐naïve group support the clinically meaningful benefits of triheptanoin in patients with LC‐FAOD. Broad inclusion criteria were employed to ensure access for patients in need with this rare disease. Although the number of patients within each group was small, the combined findings are consistent, supporting a sustained clinical benefit of triheptanoin. NBR accounts for variation in follow‐up time (attrition bias) and an ad hoc analysis of the pre‐triheptanoin data revealed no correlation between MCEs and age, eliminating maturation bias. It is unlikely that maturation resulted in such a dramatic reduction in MCEs with triheptanoin in all events across all ages and reproduced across independent populations within the study. MCEs were identified at the discretion of the physician and verified with medical review and source document verification; however, MCE severity was not captured. Across MCEs, few or no intensive care admissions were seen. Thus, robust analysis of the impact of triheptanoin on MCE severity and related outcomes such as intensive care admissions is not possible.

Finally, all assessments were identified, recorded, and analyzed in the same manner pre‐triheptanoin and with triheptanoin to mitigate assessment/detection bias. The use of objective endpoints, such as hospitalization, also reduces the risk for bias, and if anything, MCEs would be more frequently reported during the triheptanoin treatment as a result of cautious monitoring during an interventional study.^23^

In conclusion, triheptanoin is an odd‐carbon, medium‐chain triglyceride designed to replace the energy deficit and restore oxidative metabolism via the TCA cycle in patients with LC‐FAOD. The results of this study and the previous phase 2 study demonstrate that triheptanoin treatment is associated with reduced MCEs, such as rhabdomyolysis, cardiomyopathy, and hypoglycemia, as well as events leading to hospitalizations in patients with LC‐FAOD compared to pre‐triheptanoin standard of care. Triheptanoin demonstrated an acceptable safety profile, with the most common AEs being mild to moderate, transient gastrointestinal symptoms.

## CONFLICT OF INTEREST

J. C. and X. L. are an employee and shareholder of Ultragenyx Pharmaceutical Inc. J. V., B. B., G. B., N. L., J. P., A. S.‐V., K. C., P. T., S. G., and E. M. have served as a clinical investigator in clinical trials with the product manufactured by Ultragenyx Pharmaceutical Inc.

## AUTHOR CONTRIBUTIONS

**Jerry Vockley**, **Jason Cataldo**, and **Barbara Burton** participated in study design. **Jerry Vockley**, **Barbara Burton**, **Gerard Berry**, **Nicola Longo**, **John Phillips**, **Amarilis Sanchez‐Valle**, **Kimberly Chapman**, **Pranoot Tanpaiboon**, **Stephanie Grunewald**, and **Elain Murphy** participated in data acquisition. **Xiaoxiao Lu** participated in data analyses. All authors participated in data interpretation and drafting of the manuscript.

## INFORMED CONSENT

All procedures followed were in accordance with the ethical standards of the responsible committee on human experimentation (institutional and national) and with the Helsinki Declaration of 1975, as revised in 2000. Institutional review boards/ethics committees approved the protocol. Informed consent was obtained from all patients included in this study. Parents or guardians provided written informed permission for children to participate, and when appropriate, the subjects' assents were obtained before participating. Adult participants provided their own consent. An external data monitoring committee monitored patients' safety and efficacy during the study. The trial is registered with ClinicalTrials.gov (NCT02214160). Documentation of approval from the Institutional Committee for Care and Use of Laboratory Animals (or comparable committee): Institutional review boards/ethics committees, operating in accordance with Title 21 Code of Federal Regulations Part 56, approved the protocol for study UX007‐CL202 (NCT02214160).

## Supporting information


**Appendix** **S1**: Supporting information
