# Supplementary material for: Effects of triheptanoin (UX007) in patients with long‐chain fatty acid oxidation disorders: Results from an open‐label, long‐term extension study
Source: J Inherit Metab Dis. 2020 Sep 14;44(1):253–63. doi: 10.1002/jimd.12313 (PMC7891391; doi:10.1002/jimd.12313)
Supplement: Supplementary file 1 — Appendix S1: Supporting information [file JIMD-44-253-s001.docx]

# Supplementary Materials:

Figure S1. Study Design

Table S1. MCEs by Age

Psychosocial Summary Score

Additional Safety Text

UX007-CL202 Protocol

**Figure S1. Study Design**

IST, investigator-sponsored trial; SOC, standard of care

## Table S1. Total Annualized MCE Event Rates by Age

|  | **<6 yrs**  **N=14** | **6 to <18 yrs**  **N=5** | **≥18 yrs**  **N=5** |
| --- | --- | --- | --- |
| **CL201 Rollover Group** |  |  |  |
| Pre-triheptanoin, mean (SD) | 2.12 (1.21) | 1.33 (1.56) | 1.20 (2.68) |
| With-triheptanoin, mean (SD) | 1.05 (1.28) | 1.33 (1.56) | 0.60 (0.64) |
| **Triheptanoin-naïve Group** | **<6 yrs**  **N=9** | **6 to <18 yrs**  **N=7** | **≥18 yrs**  **N=4** |
| Pre-triheptanoin, median (Q1,Q3) | 1.33  (0.00, 3.65) | 2.67  (2.00, 4.00) | 3.00  (1.33, 5.67) |
| With-triheptanoin, median (Q1,Q3) | 0.00  (0.00, 1.33) | 0.91  (0.00, 1.79) | 2.21  (0.33, 9.45) |

## Psychosocial Summary Score:

The Psychosocial Summary Score was in the normal range prior to the initiation of triheptanoin for both the CL201 rollover group (mean [SD] SF-10: 50.06 [13.18], n = 7; SF-12v2: 44.59 [19.28], n = 6) and the triheptanoin-naïve group (SF-10: 45.22 [10.24], n = 7; SF-12v2: 41.54 [7.31], n = 4).

## Additional Safety Text:

There were two deaths, unrelated to treatment, in patients with the TFP LC-FAOD subtype, worsening cardiomyopathy in a 4-year-old patient in the IST/other group who had been on triheptanoin for two years and fatal cardiorespiratory arrest in a 9 month-old patient in the triheptanoin-naïve group who had been on triheptanoin for 3 months. The 4-year-old patient was hospitalized with worsening cardiac output, developed cardiac arrest, and despite resuscitation, the use of ECMO, increased diuretics, cardiac vasopressors and anticoagulants, developed ARDS, aspiration pneumonia, cardio-renal syndrome with acute kidney injury, and succumbed to extensive intracranial hemorrhage and a second, fatal cardiac arrest. The 9-month-old patient reported improvement in cardiac function at the initiation of triheptanoin, but developed mixed apnea, obstructive due to respiratory muscle hypotonia and central due to diaphragmatic weakness, leading to cardiorespiratory arrest.
